# Fine-Tuning the Amphiphilic Properties of Carbosilane Dendritic Networks towards High-Swelling Thermogels

**DOI:** 10.3390/pharmaceutics16040495

**Published:** 2024-04-03

**Authors:** Silvia Muñoz-Sánchez, Andrea Barrios-Gumiel, Francisco Javier de la Mata, Sandra García-Gallego

**Affiliations:** 1 University of Alcala, Faculty of Sciences, Department of Organic and Inorganic Chemistry, and Research Institute in Chemistry “Andrés M. Del Río” (IQAR), 28805 Madrid, Spain; silvia.munoz@uah.es (S.M.-S.); andrea.barrios@edu.uah.es (A.B.-G.); javier.delamata@uah.es (F.J.d.l.M.); 2 Networking Research Center on Bioengineering, Biomaterials and Nanomedicine (CIBER-BBN), 28029 Madrid, Spain; 3 Institute Ramón y Cajal for Health Research (IRYCIS), 28034 Madrid, Spain

**Keywords:** hydrogel, thermogel, network, polymer, dendrimer, dendron, thermo-responsive, drug delivery

## Abstract

Dendritic hydrogels based on carbosilane crosslinkers are promising drug delivery systems, as their amphiphilic nature improves the compatibility with poorly water-soluble drugs. In this work, we explored the impact of the complementary polymer on the amphiphilic properties of the dendritic network. Different polymers were selected as precursors, from the highly lipophilic propylene glycol (PPG) to the hydrophilic polyethylene glycol (PEG), including amphiphilic Pluronics L31, L35 and L61. The dithiol polymers reacted with carbosilane crosslinkers through UV-initiated thiol–ene coupling (TEC), and the resultant materials were classified as non-swelling networks (for PPG, PLU_L31_ and PLU_L61_) and high-swelling hydrogels (for PEG and PLU_L35_). The hydrogels exhibited thermo-responsive properties, shrinking at higher temperatures, and exhibited an intriguing drug release pattern due to internal nanostructuring. Furthermore, we fine-tuned the dendritic crosslinker, including hydroxyl and azide pendant groups in the focal point, generating functional networks that can be modified through degradable (ester) and non-degradable (triazol) bonds. Overall, this work highlighted the crucial role of the amphiphilic balance in the design of dendritic hydrogels with thermo-responsive behavior and confirmed their potential as functional networks for biomedical applications.

## 1. Introduction

In recent years, there has been intense research in the design of polymer networks, driven by the multiple materials accomplished like elastomers, gels and porous crystalline materials [1]. These networks, which are formed by linear polymers connected by multifunctional junctions, are highly versatile. The components (strands and junctions) can be flexible or rigid, and the connections can be covalent or non-covalent. Importantly, polymer networks represent an outstanding opportunity to control the material’s macroscopic properties at a molecular level [2]. Among polymer networks, hydrogels stand out due to their high biocompatibility, high porosity and ability to absorb large amounts of water [3,4]. They can be classified as “permanent” hydrogels, which are chemically stable and formed by covalent bonds, and “reversible” hydrogels if ionic, hydrophobic or hydrogen bonding are the main interactions within the network. Additionally, some hydrogels can exhibit stimuli-responsive properties, which are highly valuable in biomedical applications like tissue repair and drug delivery [5]. These hydrogels modify their properties in response to external stimuli like temperature. A wide variety of physical thermogels are described in the literature, based on polymers such as poly(*N*,*N*-diethylacrylamide) (PDEAAm), poly(*N*-vinylcaprolactam) (PVCL), poly[2-(dimethylamino)ethyl methacrylate] (PDMAEMA), poly(ethylene glycol) (PEG), Pluronic, and isopropylacrylamide (PNIPAM) [6]. For example, Wang and co-workers developed a dual pH/temperature-responsive hydrogel using Pluronic F127, *N*,*N*,*N*-trimethyl chitosan (TMC) and PEG-modified hyaluronic acid (PEG-HA) for their potential application in transdermal therapy [7]. The authors described an improvement in the rheological parameters in the hydrogel due to strong inter-micellar interactions. In another example, Lu et al. successfully fabricated thermo-responsive PNIPAM gels and encapsulated an antibacterial peptide, producing a sustained and controlled release over time [8]. However, most thermogels are reversible physical networks with weak bonds, so they have very poor mechanical properties and are more prone to degrade over time than covalent hydrogels [9,10].

Among the multifunctional junctions that can generate polymer networks, dendrimers are outstanding molecules due to their multivalency and monodispersity. Dendritic networks exhibit a higher control of their structure and, thus, a better structure-to-property understanding [11]. The seminal work by Gitsov and Zhu on the synthesis of amphiphilic dendritic hydrogels based on PEG and dendritic poly(benzyl ethers) [12] led to the design of many other dendritic networks based on PAMAM, PAMAM-OS, PPI, Poly-L-lysine, glycerol or polyester (bis-MPA). These include covalent networks [13], physical networks [14] and mixed inter-penetrating networks (IPN) [15].

Recently, carbosilane dendrimers have also been employed to generate dendritic hydrogels with amphiphilic properties [16,17]. The preparation of these hydrogels was carried out by click chemistry through the highly efficient thiol–ene coupling reaction (TEC). Vinyl-decorated carbosilane dendrimers reacted with a dithiol molecule in the presence of a photoinitiator and UV light, providing dendritic hydrogels that loaded and released bioactive molecules. These hydrogels were especially useful for cargoes with poor water-solubility, which lack compatibility with hydrophilic hydrogels. These studies offered some hints about the impact of the non-dendritic component. In the work by Recio-Ruiz et al. [16], DTT (a short dithiol molecule) was used, which generated low-swelling networks with an SD in the range of 6–16%. In the work by Muñoz-Sánchez et al. [17], PEG1k(SH)_2_ was used and high-swelling networks were obtained, with an SD in the range of 150–225% for traditional SiGnV_m_ dendrimers or 163–300% for the new amphiphilic dendrimers with the *N*,*N*′*-*bis(2-hydroxyethyl) ethylenediamine core.

Interested in fine-tuning the amphiphilic properties of carbosilane dendritic hydrogels, in this work, we explored the impact of the linear polymer in the generation of dendritic networks. Different polymers were selected as precursors, from the highly lipophilic propylene glycol (PPG) to the hydrophilic polyethylene glycol (PEG), including the amphiphilic Pluronics L31, L35 and L61. Pluronics are a family of block copolymers (X-Y-X) formed by polyethylene glycol (PEG) and propylene glycol (PPG). These amphiphilic copolymers are highly versatile and commercially available in a broad range of molecular weights and hydrophilic–lipophilic balance (HLB) values [18]. These polymers show remarkable property changes in response to temperature, making them very promising for biomedical applications and as a possible drug delivery vehicle due to their excellent biocompatibility and environmental sensitivity [19]. Using a similar approach through UV-initiated TEC crosslinking with carbosilane dendrimers, we accomplished either non-swelling networks or high-swelling hydrogels with thermo-responsive properties. The impact of the polymer, the pendant groups on the dendritic crosslinker and the temperature were evaluated regarding properties like the swelling degree, the drug release and the mechanical properties, as described below.

## 2. Materials and Methods

### 2.1. Materials

Reagents and solvents were purchased from commercial sources and used as received. The Pluronics L31, L35 and L61, PEG1k and PPG1k, and 3-mercaptopropionic acid and p-toluenesulfonic acid monohydrate were purchased from Sigma Aldrich (St. Louis, MO, USA). 2,2-dimethoxy-2-phenylacetophenone (DMPA) was purchased from Acros Organics (Geel, Belgium). Toluene was purchased from PanReac (Barcelona, Spain). Chloroform was purchased from J.T. Baker (Phillipsburg, NJ, USA). Acetone, tetrahydrofuran, caffeic acid and methanol were purchased from Sigma Aldrich with the HPLC grade. Sulfuric acid was purchased from Hanssell (Madrid, Spain). Vinyl-decorated dendrimer Si-G1V_8_ (**D1**) and dendrons N_3_-G3V_8_ (**D2**) and HO-G3V_8_ (**D3**) were synthesized as previously reported [20,21].

#### Synthesis of Linear Polymers P(SH)_2_

General protocol: The precursor polymer P(OH)_2_ was suspended or dissolved in toluene. Two drops of concentrated H_2_SO_4_ were added, and the solution was heated to 100 °C with stirring to obtain a clear, homogeneous solution. To this solution, 3-mercaptopropionic acid was added, and the solution was refluxed under Dean–Stark conditions for 20 h. The solvent was removed in vacuo and the polymer was isolated through different strategies.

PEG_1k_(SH)_2_ (**P1**): The polymer was prepared through the general protocol using the following reagents: PEG (1000 g/mol, 10.1 g, 10.1 mmol), toluene (100 mL), and 3-mercaptopropionic acid (3.5 mL, 40 mmol). The resultant oil was precipitated in ether, and the product was isolated as a white gel (7.4 g, 62% yield). ^1^H-NMR (CDCl_3_): δ 4.2 (t, -C*H*_2_OCO-), 3.6 (m, -O(C*H*_2_)_2_O-), 2.7 (m, -OCC*H*_2_C*H*_2_SH), 1.6 (t, -S*H*). The NMR signals matched the ones previously described in the literature [22].

PPG_1k_(SH)_2_ (**P2**): The polymer was prepared through the general protocol using the following reagents: PPG (1000 g/mol, 10.1 g, 10.1 mmol) and 3-mercaptopropionic acid (3.5 mL, 40 mmol). The resultant oil was dissolved in CH_2_Cl_2_ and washed with saturated NaHCO_3_ and NaCl. The organic phase was dried with MgSO_4_ and filtered and evaporated, obtaining the product as a yellow oil (10.5 g, 88% yield). ^1^H-NMR (CDCl_3_): δ 5.1 (t, -C*H*_2_OCO-), 3.5 (m, -OC*H*_2_C*H*(CH_3_)O-), 2.6 (m, CC*H*_2_C*H*_2_SH), 1.6 (t, -SH), 1.1 (d, -OCH_2_CH(C*H*_3_)O-).

PLU_L31_(SH)_2_ (**P3**): The polymer was prepared through the general protocol using the following reagents: Pluronic L31 (1100 g/mol, 11.7 g, 10.6 mmol) and 3-mercaptopropionic acid (3.70 mL, 42 mmol). The resultant oil was dialyzed in toluene to eliminate the impurities, obtaining the product as a yellow oil (6.0 g, 44%). ^1^H-NMR (CDCl_3_): δ 4.2 (t, -C*H*_2_OCO-), 3.6 (m, -O(C*H*_2_)_2_O-), 3.5 (m, -OC*H*_2_C*H*(CH_3_)O-), 2.6 (m, OCC*H*_2_C*H*_2_SH), 1.6 (t, -SH), 1.1 (d, -OCH_2_CH(C*H*_3_)O-).

PLU_L61_(SH)_2_ (**P4**): The polymer was prepared through the general protocol using the following reagents: Pluronic L61 (2000 g/mol, 10.3 g, 5.15 mmol) and 3-mercaptopropionic acid (1.85 mL, 21 mmol). The resultant oil was dissolved in Et_2_O and washed with saturated NaHCO_3_ and NaCl. The organic phase was dried with MgSO_4_ and evaporated, obtaining the product as a yellow oil (5.3 g, 47% yield). ^1^H-NMR (CDCl_3_): δ 4.2 (t, -CH_2_OCO-), 3.6 (m, -O(C*H*_2_)_2_O-), 3.5 (m, -OC*H*_2_C*H*(CH_3_)O-), 2.6 (m, OCC*H*_2_C*H*_2_SH), 1.6 (t, -SH), 1.1 (d, -OCH_2_CH(C*H*_3_)O-).

PLU_L35_(SH)_2_ (**P5**): The polymer was prepared through the general protocol using the following reagents: Pluronic L35 (1900 g/mol, 10.9 g, 5.75 mmol) and 3-mercaptopropionic acid (1.83 mL, 21 mmol). The resultant oil was dialyzed in toluene to eliminate the impurities, obtaining the product as a yellow oil (6.3 g, 58%). ^1^H-NMR (CDCl_3_): δ 4.23 (4 H, m, -C*H*_2_OCO-), 3.67 (4H, m, CO_2_CH_2_C*H*_2_O), 3.60 (72H, s, -O(C*H*_2_)_2_O-), 3.50 (16H, m, -OCH_2_C*H*(CH_3_)O-), 3.46 (32H, m, -OC*H*_2_CH(CH_3_)O-), 2.71 (4H, m, -OCC*H*_2_CH_2_SH), 2.62 (4H, m, -OCCH_2_C*H*_2_SH), 1.65 (2H, m, -SH), 1.10 (48H, d, -OCH_2_CH(C*H*_3_)O-). ^13^C-RMN (400 MHz CDCl_3_): ẟ 171.0 (CO_2_), 75.5 (O-*C*H-CH_3_), 75.2–73.0 (O-*C*H_2_-*C*H), 70.4 (O-*C*H_2_*C*H_2_-O), 38.9 (COO-CH_2_*C*H_2_-O), 63.5 (COO-*C*H_2_CH_2_-O), 38.5 (HS-*C*H_2_CH_2_-COO), 19.6 (HS-CH_2_*C*H_2_-COO), 17.5 (*C*H_3_-CH).

### 2.2. General Methods

NMR spectroscopy. NMR spectra were acquired at CAIQ-UAH, using the Varian NMR System-500 (Varian, Inc., Palo Alto, CA, USA), Varian Mercury Plus-300 and Bruker AVANCE Neo 400 instruments (Bruker, Billerica, MA, USA) at room temperature and using CDCl_3_ as the solvent. The chemical shifts are expressed in ppm using the solvent as an internal reference in ^1^H-NMR (CDCl_3_ δ (H) = 7.24 ppm) and in ^13^C-NMR (CDCl_3_ δ (C) = 77.0 ppm). HSQC was also carried out under these conditions.

Mass spectrometry. A MALDI-TOF/TOF, model ULTRAFLEX III of BRUKER, was used. The sample was dissolved in dichloromethane (2 mg/mL). The sample was mixed with the DCTB matrix (8 mg/mL in DCM) and NaI (2 mg/mL in acetone). The detection was performed in reflector mode with the detection of positive ions in the range of 500–5000 Da.

High-Performance Liquid Chromatography (HPLC). Drug release studies were performed at CAIQ-UAH on Agilent 1200 HPLC equipment (Agilent, Santa Clara, CA, USA), using an ACE Excel 5 C18 250 × 4.6 mm column and a mobile phase of 2% acetic acid and acetonitrile (50:50), with an injection volume of 10 μL. The drug was detected at a wavelength of 254 nm.

Rheometer. The viscoelastic properties of hydrogels were measured using a Discovery Hybrid Rheometer 10 (DHR-10) from TA Instruments (New Castle, DE, USA) at 25 °C and 37 °C using parallel-plate geometry (8 mm diameter). An amplitude sweep (0.1–100% strain at 1 Hz), a frequency sweep (0.1–10 Hz at 1% strain) and ramp temperature (20–40 °C at 1 Hz and 1% strain) assays were performed.

### 2.3. Synthesis of Dendritic Hydrogels

#### 2.3.1. Synthesis of Dendritic Hydrogels

P(SH)_2_ (4 eq.) was dissolved in a THF:MeOH mixture (1:2, 150 μL), and subsequently, the dendrimer/dendron (1 eq.) and DMPA (5 mol% of vinyl groups) were added. The resulting mixture was argon bubbled for a few seconds and introduced into several Teflon plugs with a capacity of around 200 μL before exposure to UV light for 4–6 h. A UV darkroom Vilber CN-15.LC was used (Vilber, Eberhardzell, Germany), with a total intensity of 30 W at 365 nm. Finally, the hydrogels were washed several times with acetone until the DMPA was eliminated, as confirmed by TLC (hexane:ethyl acetate, 80:20).

#### 2.3.2. Post-Functionalization of Dendritic Hydrogels

Esterification of Rhodamine B to *Hy*[(HO-G3V_8_)x(PLU_L61_(SH)_2_)]. Rhodamine B (1.2 eq.) was dissolved in dichloromethane (4 mL) and was reacted with EDCI·HCl (1.2 eq.). The mixture was stirred for 30 min, and DMAP (0.2 eq.) was added. Subsequently, the hydrogel (70 mg) was immersed in the solution and maintained with slight stirring (150 rpm). After 20 h, the hydrogel was thoroughly washed with dichloromethane to remove non-bound dye, and was dried, obtaining a pink hydrogel.

CuAAC covalent attachment of Rhodamine B to *Hy*[(N_3_-G3V_8_)x(PLU_L61_(SH)_2_)]. Rhodamine B isothiocyanate (50 mg, 0.093 mmol) was reacted with propargylamine (6 µL, 0.093 mmol) to generate the alkyne derivative of Rhodamine B. Subsequently, an aqueous solution of the alkyne derivative of Rhodamine (1.2 eq) was prepared. Copper sulfate (1.2 eq., 2.8 mg, 0.011 mmol) and sodium ascorbate (2.4 eq., 4.5 mg, 0.0226 mmol) were added. The hydrogel was immersed in the solution and maintained at 50 °C, with slight stirring (150 rpm) for 20 h. The hydrogel was thoroughly washed with water and acetone to remove non-bound dye, and was dried, obtaining a pink hydrogel.

### 2.4. Characterization of Hydrogels

#### 2.4.1. Swelling Degree (SD%)

The hydrogels were immersed in distilled water for several days. Periodically, they were removed, and their size and weight were measured. All gels were tested in duplicate. The SD is calculated with the following equation:

SD% = ((W − W_D_)/W) × 100

where W is the weight of the swollen gel, and W_D_ is the dry gel mass after purification.

#### 2.4.2. Crosslinking Degree (CD%)

The crosslinking percentage was calculated using the following equation:

CD(%) = (1 − (M_UV_ − M_W_)/M_UV_) × 100

where W_UV_ is the dry mass after the UV reaction, and M_W_ is the dry mass after purification. All measurements were performed in duplicate.

#### 2.4.3. Caffeic Acid Loading and Release Assay

The drug loading was performed by immersing the dry hydrogel (~60 mg) in a solution of caffeic acid (50 mg in 1 mL ethanol) for 60 h at 37 °C. Then, the hydrogel was extracted from the solution and dried to remove all embedded solvents. The remaining caffeic acid in the ethanol solution was quantified through HPLC.

For the release assay, the CA-encapsulated hydrogel was immersed in 1 mL of water solution at either 25 °C or 37 °C. Small aliquots (50 µL) were collected over time to quantify the CA release through HPLC. The solvent volume was kept constant by adding 50 µL of water after each extraction, so a small dilution effect (5% decrease in concentration) must be considered.

## 3. Results

Dendritic hydrogels based on carbosilane crosslinkers are promising materials as drug delivery systems. In our previous studies, different vinyl-decorated dendrimers were reacted with DTT or PEG(SH)_2_ (**P1**) through TEC click chemistry, delivering amphiphilic networks that improved compatibility with lipophilic drugs. Additionally, we demonstrated their versatility and the ability to respond to certain stimuli like pH and temperature. In this work, we explored the impact of the linear polymer on the design of amphiphilic hydrogels.

### 3.1. Synthesis of Linear Polymers

In the design of the amphiphilic hydrogels, we used a lipophilic carbosilane crosslinker and a complementary hydrophilic polymer. To analyze the required hydrophilic-lipophilic balance to generate adequate hydrogels, we synthesized a library of different polymers P(SH)_2_ from different precursors: PEG_1k_, PPG_1k_, PLU_L31_, PLU_L35_ and PLU_L61._ We adapted the protocol previously described for **P1** by Macdougall et al. [18]. The dithiol polymers (**P1**–**P5**) were prepared from the commercial precursors through esterification with 2-mercaptopropionic acid at 100° C in toluene. Depending on the nature of the polymer, different isolation protocols were employed, which impacted the final yield. Moderate yields were achieved in the range of 45–90%, being higher for the most lipophilic polymer. The final polymers were characterized through NMR, confirming the presence of the new -SH groups required for the crosslinking (Figures S1–S4). The polymers presented different HLB values, from the highly hydrophilic **P1** (HLB 18) to the highly hydrophobic **P2** (HLB 10.78).

**Figure 1:**
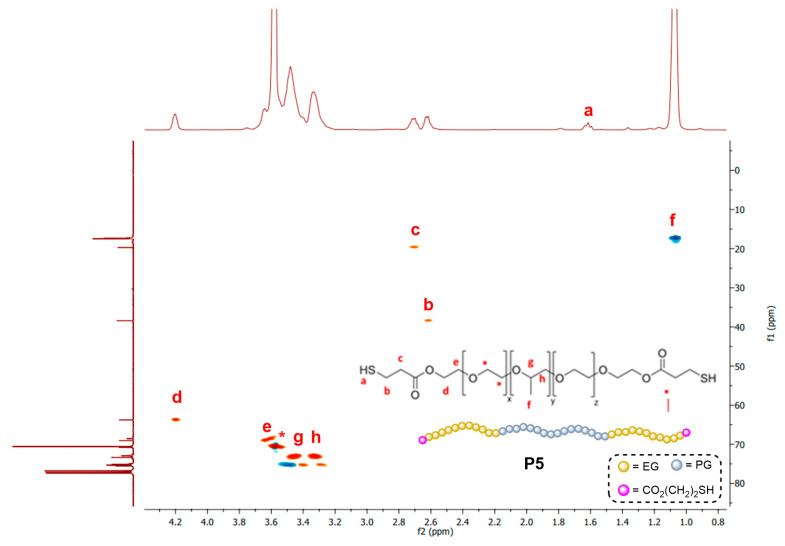
HSQC{^1^H-^13^C} spectrum of PLU_L35_(SH)_2_ (**P5**) in CDCl_3_, confirming the presence of the -CO(CH_2_)_2_SH fragment in the terminals of the polymer. ^1^H-NMR spectrum is shown on the top, and ^13^C-NMR is shown on the left of the figure.

The characterization of **P5** is discussed as an example. The HSQC spectrum is presented in Figure 1, while the individual ^1^H and ^13^C spectra are included in Figure S1. Compared to the precursor polymer, **P5** showed three new multiplets in the ^1^H-NMR spectrum at 1.65, 2.62 and 2.71 ppm from the -CO(CH_2_)_2_SH fragment (signals a, b and c in Figure 1). Furthermore, the methylene group closer to the new ester bond (signal d) shifted to 4.2 ppm. The methylene groups from the ethylene glycol (EG) units (labeled as *, e) and those from the propylene glycol (PG) units (labeled as f, g, h) exhibit much less shifting. In ^13^C-NMR, a signal around 171.0 ppm is observed, assigned to the carbonyl group. This polymer was also studied through MALDI-TOF. A Gaussian curve is observed between 700 and 3500 Da, centered around 2200 Da (Figure S5). Despite the complexity of the spectra, due to the variable number of EG and PG units, it confirmed the correct attachment of both terminal thiol units.

### 3.2. Synthesis and Characterization of Dendritic Hydrogels

**Figure 2:**
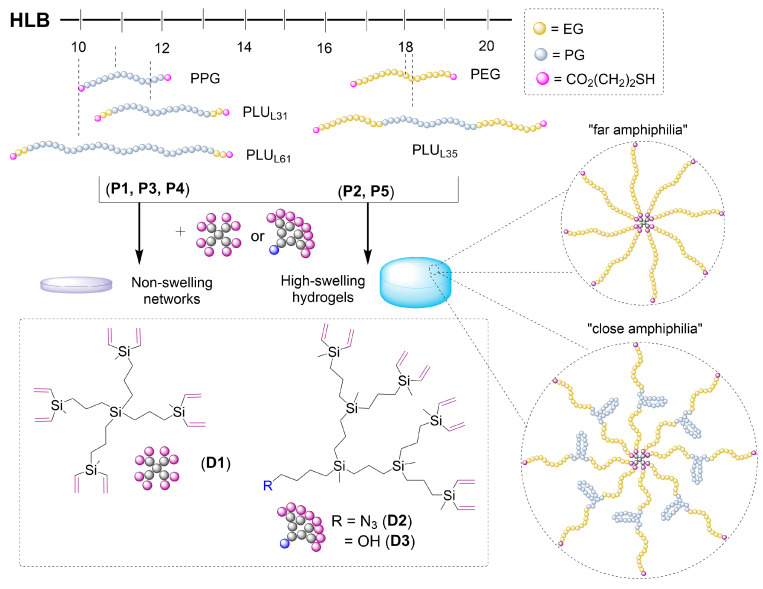
Schematic overview of the synthesis of the dendritic networks. Top: Polymer precursors **P1**–**P5**, including their HLB. Bottom: Dendritic precursors **D1**–**D3**. Middle: Resultant networks and hydrogels, including representation of their nanostructuring in water. In “far” amphiphilia, the hydrophobic and hydrophilic sections are provided by different components (dendrimer and PEG, respectively), so the hydrophobic pockets are located more distantly in the hydrogel. In “close” amphiphilia, the polymer is intrinsically amphiphilic (Pluronic L35), and the hydrophobic pockets caused by the dendrimer and the PPG sections are found closer.

For the synthesis of the dendritic hydrogels, we followed the recently reported protocol using a vinyl-decorated carbosilane dendritic crosslinker and a dithiol linear polymer; Figure 2 [16,17]. Photoinitiated thiol–ene coupling (TEC) was used as the main tool. In a general protocol, the dendritic crosslinker and the polymer were dissolved in a THF:MeOH mixture. Subsequently, DMPA (5 mol% alkene) was added, and the mixture was exposed to UV light (365 nm) until the hydrogel was fully cured. Finally, the hydrogel was washed with acetone until complete removal of the photoinitiator and other impurities was achieved. All the hydrogels were characterized by studying their crosslinking degree (CD%) and swelling degree (SD%) in water.

#### 3.2.1. Impact of the Linear Polymer P(SH)_2_

**Figure 3:**
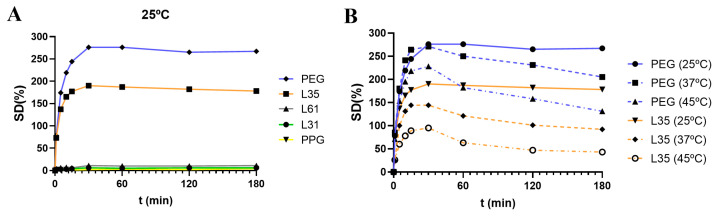
(**A**) Comparative swelling degree at 25 °C of hydrogels *Hy*[(Si-G1V_8_)x(PX)], prepared from carbosilane dendrimer **D1** and the different polymers. (**B**) Comparative swelling degree at 25, 37 and 45 °C of hydrogels based on PEG_1k_ and PLU_L35_.

In the first experiment, we performed a screening of the different linear polymers P(SH)_2_ to evaluate the impact on the swelling properties of the dendritic networks. For comparative purposes, we used dendrimer Si-G1V_8_ (**D1**) for the synthesis of all networks and studied their swelling degree at 25 °C (Figure 3). As expected, the network prepared with the hydrophobic PPG did not swell, and the one prepared from the hydrophilic PEG swelled up to 275% (at 25 °C); for the amphiphilic Pluronics, with intermediate properties between PPG and PEG, a different behavior was found. PLU_L31_ and PLU_L61_ are short polymers (M_n_ 1100 and 2000, respectively) with only 10% PEG. The crosslinking with the highly lipophilic dendrimer **D1** generates a network incapable of swelling in water (Figure 3A). However, for PLU_L35_ (M_n_ 1900 and 50% PEG), the hydrogel swells up to 190%. These results highlight the impact of the hydrophilic–lipophilic balance within the network to accomplish an effective swelling.

Overall, we found two dendritic hydrogels with an adequate amphiphilic balance to swell in water: *Hy*[(Si-G1V_8_)x(P1)] (**H1Si**), with “far amphiphilia”, as the lipophilic and the hydrophilic properties are provided by different entities, and thus the hydrophobic pockets are located more distantly in the hydrogel; and *Hy*[(Si-G1V_8_)x(P5)] (**H5Si**), with “close amphiphilia”, due to the presence of an intrinsically amphiphilic polymer in the network, and then the hydrophobic pockets caused by the dendrimer and the PPG fragments are found closer. The swelling behavior of these two hydrogels was further explored at different temperatures (Figure 3B). A similar pattern is observed: a fast swelling in the first 30 min to reach a maximum, then a progressive decrease until reaching a plateau around 2 h. In both cases, the swelling decreased when increasing the temperature. At higher temperatures, the higher mobility of the polymer chains enables a collapse of the lipophilic sections and expels the molecules of water from the pores of the network.

#### 3.2.2. Impact of the Dendritic Crosslinker

In the second round of experiments, we selected two polymers with similar lengths but very different natures: the hydrophobic PLU_L61_(SH)_2_ (**P4**) and the hydrophilic PLU_L35_(SH)_2_ (**P5**); and we also modified the dendritic crosslinker: Si-G1V_8_ (**D1**), N_3_-G3V_8_ (**D2**) and HO-G3V_8_ (**D3**). The crosslinkers presented the same number of alkene groups available for crosslinking but had a different topology, having azide or hydroxyl groups in the focal points of the dendrons. This assay provided information about the impact of topology and pendant groups in two different types of dendritic networks: non-swelling (using **P4**) and high-swelling (using **P5**). The polymers (4 eq.) were crosslinked with **D1**–**D3** (1 eq.) to generate the corresponding networks *Hy*[(X-GnV_8_)x(P4)] and *Hy*[(X-GnV_8_)x(P5)] (Table 1).

**Figure 4:**
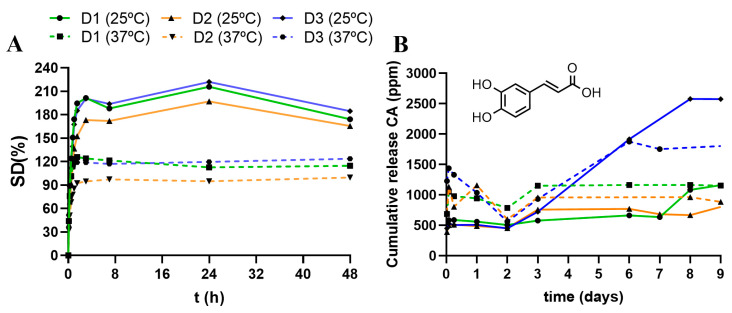
Comparative swelling degree (**A**) and cumulative release of caffeic acid (**B**) at 25 and 37 °C of *Hy*[(X-GnV_8_)x(PLU_L35_)] hydrogels, with Si-G1V_8_ (**D1**), N_3_-G3V_8_ (**D2**) and HO-G3V_8_ (**D3**).

As expected, none of the PLU_L61_ networks swelled in water. For PLU_L35_ hydrogels, the SD% increased with a decreasing temperature, following the same pattern in both tests (25 and 37 °C); Figure 4A. The non-functional and the HO-functional hydrogels (with **D1** and **D3** crosslinkers, respectively) showed a similar SD of 200–220% at 25 °C, which substantially decreased to 95–120% at 37 °C. Both have a higher swelling than the N_3_-functional hydrogel (with **D2** crosslinker) despite having a higher CD%. This fact highlights the impact of the pendant group on the ability to swell in water.

The impact of the dendritic crosslinker was also studied regarding the drug loading and release of hydrophobic cargo. As a proof-of-concept, caffeic acid was loaded in the hydrogels, and the release was studied over time at 25 and 37 °C. The loading was performed by immersing the dry hydrogels in a solution of caffeic acid (50 mg in 1 mL of ethanol) for 60 h at 37 °C. These conditions were selected to maximize the loaded cargo. Then, the hydrogels were removed from the solution and dried to remove all embedded solvents. The remaining caffeic acid on the ethanol solution was quantified through HPLC, showing that the hydrogels loaded 25.5 ± 0.9 mg, 23.9 ± 3.4 mg and 19.3 ± 1.4 mg for **H5OH**, **H5N_3_** and **H5Si**, respectively. Accordingly, the loading efficiency is in the range of 39–51%, which is positively influenced by the presence of the pendant groups.

CA-encapsulated hydrogels were then immersed in a water solution at 25°C or 37°C. Small aliquots (50 µL) were collected over time to quantify the CA release through HPLC. The results are represented in Figure 4B. At 25 °C, the three hydrogels behaved similarly for the first 48 h, showing no release. However, after 48 h, relevant differences appeared, showing a release that follows the trend Si (**D1**) < N_3_ (**D2**) << OH (**D3**). At 37 °C, a higher release was observed compared to 25 °C. **H5Si** follows a similar pattern at both temperatures but duplicates the amount of released CA. The maximum released amount was accomplished around day 3 at 37 °C, but required 9 days at 25 °C to reach the same amount. **H5N_3_** exhibited a burst release during the first hours, but the cargo was re-encapsulated during the first 2 days. Then, a sustained release started, reaching a maximum amount around day 3, which continued until day 8. **H5OH** also exhibited a burst release and a re-encapsulation of cargo during the first 2 days and then a subsequent sustained release until day 6. This assay confirmed the impact of both the pendant groups and the temperature to modulate the drug release.

This initial drug resorption had already been observed in other carbosilane dendritic hydrogels [12]. The hydrogel prepared with the traditional dendrimer Si-G1V_8_ exhibited a burst release of ibuprofen and then a sustained release during the first 4 days. However, the hydrogels prepared with the aromatic-core dendrimer ArG2V_12_ exhibited a resorption of the drug during the first 3 days and then a subsequent sustained release. When immersed in water, the hydrogel underwent a redistribution of its nanostructure, with a potent “sponge effect”, which later acted as a reservoir of the aromatic drug. In the present work, the CA resorption occurs during the first 2 days, probably related to the rearrangement of PLU_L35_ chains in water. The change of solvent from the loading step (ethanol) to the release step (water) requires some time to equilibrate the encapsulated cargo in the hydrogels. This resorption is especially relevant in the presence of azide and hydroxyl pendant groups, which aid in CA capture.

### 3.3. Mechanical Characterization of Dendritic Hydrogels

Rheology provides quantitative information on the viscoelasticity of the hydrogels. We herein explored hydrogels *Hy*[(X-GnV_8_)x(PLU_L35_)] with **D1** (X = Si), **D2** (X = N_3_) and **D3** (X = OH) to evaluate the impact of the pendant groups in a non-swelling state. Two main parameters were studied: the storage modulus (G′), which measures the elasticity, and the loss modulus (G″), which represents the viscous part of a material and has the ability to dissipate energy in the form of heat. If G′ > G″, the material can be considered mainly elastic.

**Figure 5:**
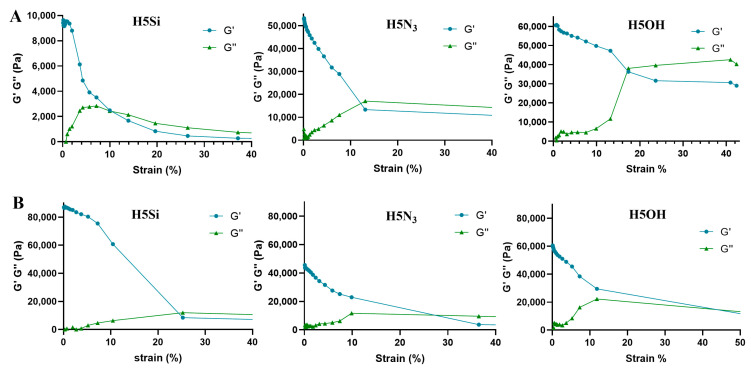
Amplitude sweeps at 25 °C (**A**) and 37 °C (**B**) for each hydrogel **H5X**.

The measurements must be performed within the linear viscoelastic region (LVR), where the material can be deformed without losing its microstructural properties. We calculated the LVR through an amplitude sweep assay at 25 and 37 °C, keeping the frequency at 1 Hz and varying the oscillatory strain from 0.1% to 100%. A sudden drop in G′ that ultimately intersects with G″ indicates the critical strain, where the material microstructure is altered and, therefore, its viscoelastic properties. As depicted in Figure 5, the temperature is crucial for the critical deformation. At higher temperatures, the hydrogel can tolerate higher strains before being permanently deformed. For **H5Si**, the critical strain switches from 10% (25 °C) to 25% (at 37 °C). For **H5N_3_**, it changes from 12% to 27%, and for **H5OH**, from 17% to 45%. Accordingly, we selected 1% OS as the optimum.

**Figure 6:**
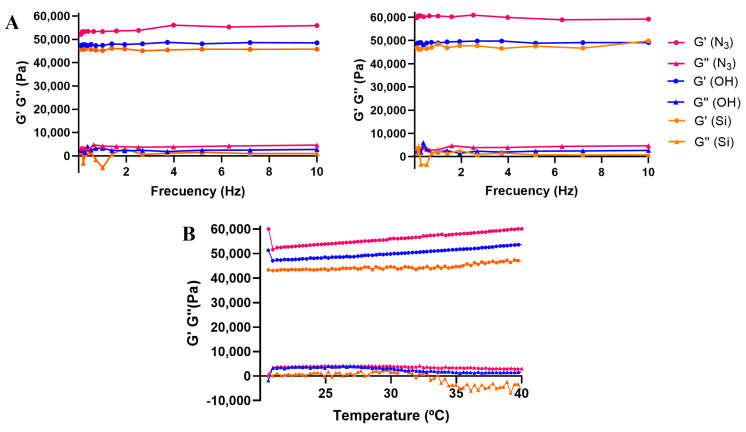
Storage (G′) and loss (G″) moduli in (**A**) frequency sweeps at 25 °C (**left**) and 37 °C (**right**), with OS 1%; (**B**) temperature sweep, from 20 to 40 °C, at 1 Hz and 1% strain; for hydrogels *Hy*[(X-GnV_8_)x(P5)].

Once the LVR was established, the hydrogels were studied through a frequency sweep assay. The assay was performed in the range of 0.1–10 Hz with the OS at 1% at 25 and 37 °C; Figure 6A. This assay provides more information about the behavior of the material (solid, liquid or gel). Due to the gel nature of our materials, they present a phase angle (δ) independent of the frequency and, therefore, a horizontal linearity. The storage moduli are substantially higher than the loss moduli, confirming the elastic (solid-state) nature of the hydrogels. At both temperatures, all hydrogels were stable, as G′ and G″ were not affected by the increase in frequency. Commonly, the storage modulus increases with the crosslinking degree of the hydrogel. However, we herein observed that the storage moduli decreased **D2** (N_3_) > **D3** (OH) > **D1** (Si), in an opposite trend to the CD%. These results highlight the impact of the pendant groups, which reinforce the elasticity of the network.

In a final assay, we performed a temperature sweep in the range of 20–40 °C at a constant frequency and deformation (1 Hz and 1% strain). As shown in Figure 6B, all hydrogels followed a similar trend, slightly increasing the storage modulus (G′) with the temperature and visibly decreasing the loss modulus (G″) above 35 °C, especially in **D1**. This indicates that the hydrogels are rheologically stable in the range of 20–40 °C and that the solid-state behavior is reinforced at higher temperatures.

### 3.4. Post-Functionalization of Dendritic Networks

The dendritic networks prepared with crosslinkers **D2** and **D3** present available azide and hydroxyl groups for further post-functionalization. As a proof-of-concept, we studied the covalent attachment of the fluorescent tag rhodamine through degradable and non-degradable bonds. In this case, we used the non-swelling networks *Hy*[(HO-G3V_8_)x(PLU_L61_(SH)_2_)] (**H4OH**) and *Hy*[(N_3_-G3V_8_)x(PLU_L61_(SH)_2_)] (**H4N_3_**) as precursors to explore the accessibility of the reactants in non-swelling situations.

**Figure 7:**
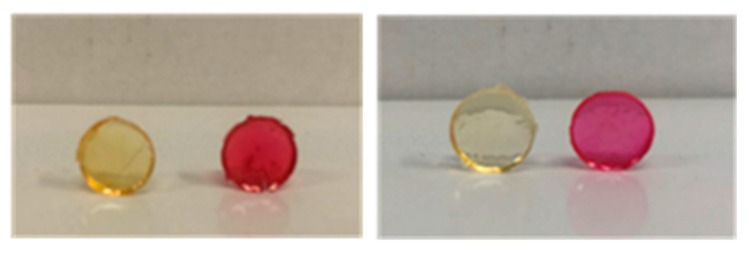
Image depicting hydrogels Hy[(HO-G3V_8_)x(PLU_L61_(SH)_2_)] (**H4OH**) and Hy[(N_3_-G3V_8_)x(PLU_L61_(SH)_2_)] (**H4N_3_**) before (yellow color) and after (pink color) covalent attachment of Rhodamine B.

On the one hand, we performed the esterification of Rhodamine B with EDCI·HCl in dichloromethane for 20 h at r.t. After thorough washings with dichloromethane to remove any unreacted compound, we visually confirmed the attachment of rhodamine (Figure 7). On the other hand, we performed a CuAAC click reaction on the azide bonds to generate permanent triazol bonds in the network. We first modified the Rhodamine B isothiocyanate with propargylamine. Then, we performed the CuAAC reaction in water in the presence of CuSO_4_ and sodium ascorbate for 20 h at 50 °C. Again, after thorough washings with water and acetone, we visually confirmed the attachment of rhodamine. This result supports the ability of the dendritic networks to undergo post-modification with relevant moieties, like fluorescent tags, drugs or other bioactive molecules.

## 4. Discussion

The field of polymer networks pursues a key target: to control the material’s macroscopic properties at a molecular level. In this work, we contribute to this target by addressing three relevant elements: linear polymers, multifunctional junctions and crosslinking reactions.

Dendritic crosslinkers can be seen as a relevant tool due to their multivalent but defined structure that improves structural control. Additionally, their nature, size and functionalities can be fine-tuned, being highly versatile. In particular, we herein demonstrated that carbosilane dendrimers offer unique properties as multifunctional crosslinkers arising from their monodisperse, highly stable and lipophilic properties. Unlike other dendrimers employed in dendritic networks, with a main hydrophilic nature, carbosilane systems are especially interesting for the design of amphiphilic hydrogels for drug delivery purposes, as they increase the compatibility with poorly water-soluble drugs. Additionally, they can be precisely defined to incorporate pendant groups in the networks using heterofunctional dendrons like N_3_-G3V_8_ (**D2**) and HO-G3V_8_ (**D3**).

Regarding the linear polymers, in this work, we demonstrated the crucial impact of the amphiphilic balance by switching the nature of the linear polymer. An adequate amphiphilic balance delivered high-swelling hydrogels, while a mainly hydrophobic framework generated non-swelling networks. Two different types of hydrogels were accomplished as follows: with “far” amphiphilia, where the hydrophobic and hydrophilic sections are provided by different components (dendrimer and PEG, respectively), or with “close” amphiphilia, as the used polymer is intrinsically amphiphilic (Pluronic L35). Despite the fact that PLU_L35_ is longer than PEG_1k_, the PEG hydrogels exhibited higher swelling, confirming the impact of the linear counterpart. The modification of Pluronic polymers with terminal hydrophobic units affects their sol–gel transition. Qian et al. designed a thermosensitive hydrogel based on PCL-PLU_L35_-PCL [23]. This polymer exhibited a special gel–sol transition arising from the hydrophobic interactions and partial crystallization. However, if the molecular weight of the PCL portion reaches an excessive value, it becomes too hydrophobic for thermo-reversible gelation. This example also supports the impact of amphiphilic balance on the design of polymer networks.

Finally, the use of highly efficient crosslinking reactions like thiol–ene chemistry (TEC) can facilitate the structural control of the networks. TEC has been previously employed to generate different dendritic networks, through a fast and robust approach [24,25]. In our case, TEC generated a high CD% (70–95%), which also depended on the lipophilicity of the polymer and the dendritic pendant groups. The more lipophilic network, the higher the crosslinking degree was observed. Other photo-cross-linkable hydrogels have been described in the literature, mainly relying on modified polymers [26]. For example, Pluronic F127 was di-acrylated and crosslinked upon exposure to UV light in the absence and presence of vinyl-functional hyaluronic acid, which acted as a multifunctional crosslinker [27]. The chemical crosslinking resulted in a micellar hydrogel network structure. Similarly to our studies, the authors demonstrated that at higher temperatures (37 °C vs. 13 °C), swelling ratios substantially decreased. They also observed that the SD% continuously decreases with increasing temperature until a sharper decrease around 20 °C [28]. This indicated that the local micellization of the Pluronic component had occurred, which was induced by rising temperature, and played a critical role in the de-swelling of the HA/Pluronic hydrogels. The hydrophobic PPG middle block of Pluronic self-associated and reduced the mesh size in the hydrogels at higher temperatures. Nevertheless, the authors also described the mass erosion of these hydrogels. The micelles slowly degraded due to the hydrolytic scission of the ester linkage between the Pluronic and acrylate groups. Accordingly, the use of thiol–ene chemistry as herein reported, offers a higher stability to the covalently crosslinked Pluronic hydrogels.

These three components, linear polymer, dendritic crosslinker and the TEC reaction, clearly affected the design of the networks as well as their properties, like swelling, drug release and mechanical properties. The swelling was mainly determined by the amphiphilic balance, requiring overcoming a certain barrier to jump from a non-swelling network to a high-swelling hydrogel. The PEG_1k_ and PLU_L35_ polymers enabled us to overcome this barrier. Subsequently, the type of amphiphilia (“far” or “close”) fine-tuned the swelling behavior of the network, as well as the thermo-responsive behavior of the hydrogel. These hydrogels showed a lower swelling at 37 °C than at 25 °C due to the rearrangement of the hydrophobic sections in the network and the nanostructuring that expelled the water molecules from the hydrogel. The nanostructuring of hydrogels has been extensively described in the literature. For example, amphiphilic block copolymers and peptide oligomers with specific sequences can go through self-assembly to form a nanostructured hydrogel under physiological conditions [27]. Composite hydrogels have also been described, which include metal nanoparticles, carbon nanotubes, nano-cellulose or MOFs [29]. These embedded nanomaterials can modulate the biological response. The drug release, herein exemplified with caffeic acid, is closely connected to the swelling. At 37 °C, the drug release is higher. This behavior can also be modulated by the presence of pendant groups: the hydroxyl groups substantially improve the release. Furthermore, a peculiar pattern was found at 37 °C, exhibiting a burst release, then drug resorption for the first 48 h, and a subsequent sustained release. These hydrogels showed a restructuring of the nanostructure upon exposure to water, which acted as a drug reservoir.

The mechanical properties of our hydrogels, with G′ >> G″ over the entire frequency range, are in agreement with an extremely soft viscoelastic solid. This behavior has already been seen for other photo-crosslinked Pluronic hydrogels, with G′ values in the range of 1000–10,000 Pa [27,30,31]. For our hydrogels, much stiffer networks were obtained above 45,000 Pa. All these examples confirmed that G′ and G″ were much higher than that of the physical Pluronic hydrogels produced by simply increasing the temperature [32]. For physical Pluronic hydrogels, with the temperature rise, the hydrophobic interactions inside the Pluronic increased and determined the micelles and polymicelles formation, which further generated a physical network. After the transition point, G′ becomes higher than G″, suggesting network formation. With our hydrogels, we also confirmed that the storage modulus was clearly affected by the pendant groups, showing the following trend: **D2** (N_3_) > **D3** (OH) > **D1** (Si). Surprisingly, this was an inverse trend to the CD%. This fact revealed the potential reinforcement of the covalent network with additional crosslinking points due to, for example, H-bonding.

## 5. Conclusions

Carbosilane-based dendritic hydrogels enable good control of the material macroscopic properties at a molecular level. The use of versatile linear polymers, monodisperse multifunctional crosslinkers and a highly efficient crosslinking reaction like TEC improve the control over the design of the network. The precise design enables the achievement of on-demand properties, like adequate hydrophilic–lipophilic balance, to obtain high-swelling hydrogels with thermo-responsive properties. These networks can be designed with “far” or “close” amphiphilia, which impacts the hydrogel nanostructuring and thus fine-tunes their properties like swelling and drug release. Furthermore, they can present pendant groups that can be post-functionalized to provide additional properties. This opens new avenues for different biomedical applications that require permanent activity on the network or even a more controlled release of a molecule through covalent bond cleavage. Overall, this work highlighted the potential of carbosilane dendritic hydrogels with thermo-responsive behavior in the biomedical field.

## 6. Patents

Some of the results of this article are included in the patent “Hidrogeles con dominios dendríticos de naturaleza carbosilano, su preparación y sus usos”, PCT/ES2022/070728.

## Figures and Tables

**Table 1:** Overview of the different linear polymers and the derived dendritic hydrogels, including relevant parameters.

Polymer	M_n_^a^ (g/mol)	%PEG	HLB ^b^	Dendritic Hydrogel	CD (%)	SD (%) ^c^ 25/37 °C
PEG_1k_(SH)_2_ (**P1**)	1000	100	18	*Hy*[(Si-G1V_8_)x(P1)] (**H1Si**)	91 (3 h)	276/271
PPG_1k_(SH)_2_ (**P2**)	1000	0	10.78	*Hy*[(Si-G1V_8_)x(P2)] (**H2Si**)	94 (3 h)	2/3
PLU_L31_(SH)_2_ (**P3**)	1100	10	11.80	*Hy*[(Si-G1V_8_)x(P3)] (**H3Si**)	94 (3 h)	6/5
PLU_L61_(SH)_2_ (**P4**)	2000	10	10.05	*Hy*[(Si-G1V_8_)x(P4)] (**H4Si**)	71 (3 h)	11/9
				*Hy*[(N_3_-G3V_8_)x(P4)] (**H4N_3_**)	92 (3 h)	^d^
				*Hy*[(HO-G3V_8_)x(P4)] (**H4OH**)	92 (3 h)	^d^
PLU_L35_(SH)_2_ (**P5**)	1900	50	18.10	*Hy*[(Si-G1V_8_)x(P5)] (**H5Si**)	90 (4 h)	216/112
				*Hy*[(N_3_-G3V_8_)x(P5)] (**H5N_3_**)	76 (4 h)	198/95
				*Hy*[(HO-G3V_8_)x(P5)] (**H5OH**)	81 (4 h)	222/120

^a^ Calculated M_n_ of precursor polymer. ^b^ Calculated through Davies method (ChemAxon). ^c^ Maximum swelling degree. ^d^ Not measured.

## Data Availability

The data presented in this study are available in this article (and Supplementary Material).

## Supplementary Materials

The following supporting information can be downloaded at: https://www.mdpi.com/article/10.3390/pharmaceutics16040495/s1, Figures S1–S4: ^1^H, ^13^C-NMR of polymers P2–P5; Figure S5: MALDI-TOF spectra of polymer P5.
