# Supplementary material for: Fine-Tuning the Amphiphilic Properties of Carbosilane Dendritic Networks towards High-Swelling Thermogels
Source: Pharmaceutics. 2024 Apr 3;16(4):495. doi: 10.3390/pharmaceutics16040495 (PMC11054174; doi:10.3390/pharmaceutics16040495)
Supplement: Supplementary file 1 [file pharmaceutics-16-00495-s001.zip › pharmaceutics-2920725-supplementary.pdf]

Electronic Supporting Information

Fine-tuning the amphiphilic properties of carbosilane dendritic networks towards high-swelling thermogels

Silvia Muñoz-Sánchez,[a] *Andrea Barrios-Gumiel,*[a] *F. Javier de la Mata* [a,b,c] *and Sandra García-Gallego** [a,b,c]

a. University of Alcala, Department of Organic and Inorganic Chemistry and Research Institute in Chemistry "Andrés M. Del Río" (IQAR), 28805, Madrid, Spain. silvia.munoz@uah.es

b. Networking Research Center on Bioengineering, Biomaterials and Nanomedicine (CIBER-BBN), 28029, Madrid, Spain;

c. Institute Ramón y Cajal for Health Research (IRYCIS), 28034, Madrid, Spain.

Table of contents.

Figure S1. ^{1}H and ^{13}C NMR spectra of polymer **P5**……………………………………………………………….……2

Figure S2. ^{1}H NMR spectrum of polymer **P2**…………………….…………………………………………………….……..3

Figure S3. ^{1}H NMR spectrum of polymer **P3**…………………….…………………………………………………….……..3

Figure S4. ^{1}H NMR spectrum of polymer **P4**…………………….…………………………………………………….……..3

Figure S5. MALDI spectrum of polymer **P5**……………..………….…………………………………………………….……..4

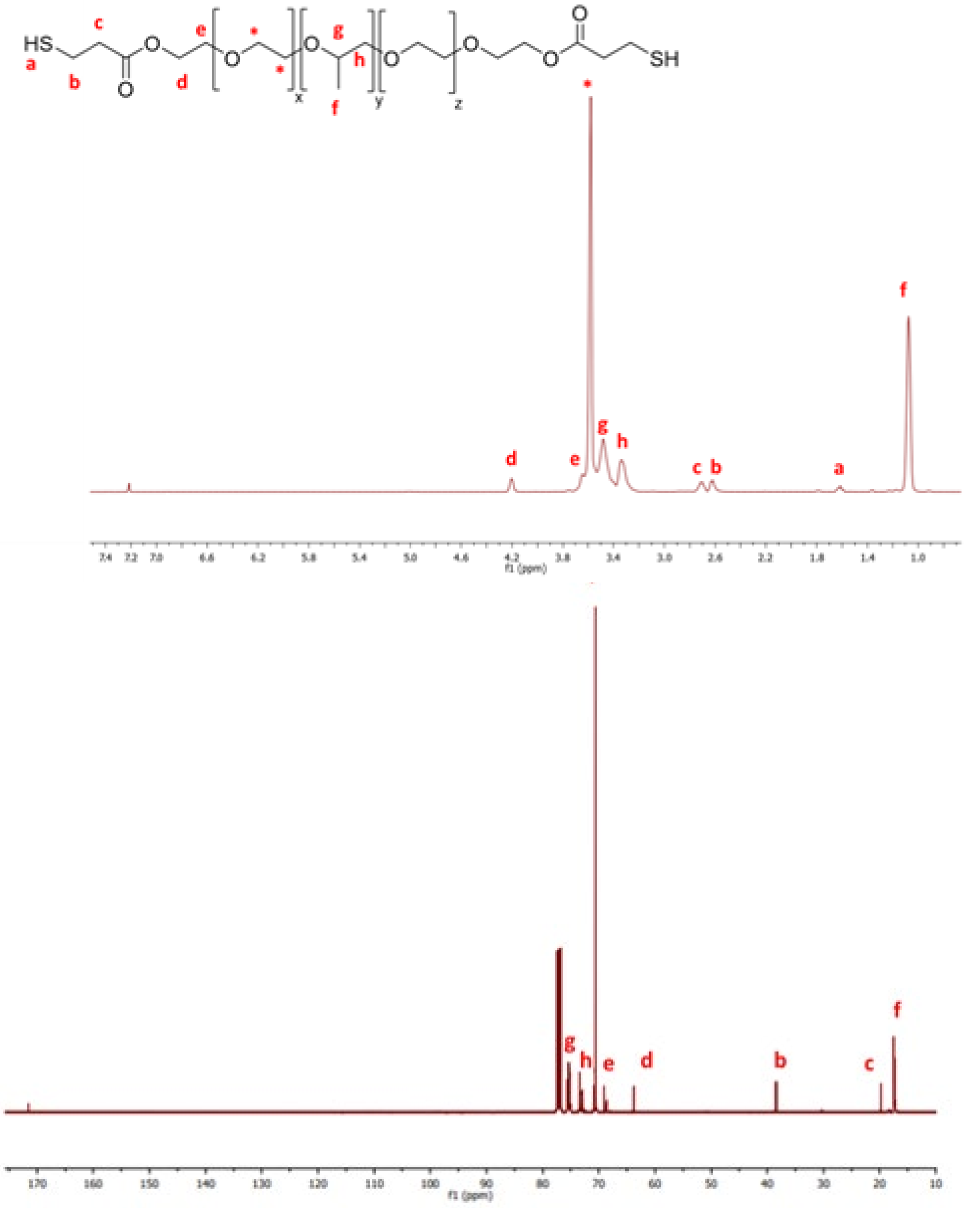

Figure S1. ^{1}H and ^{13}C NMR spectra of polymer **P5** in $CDCl_3$.

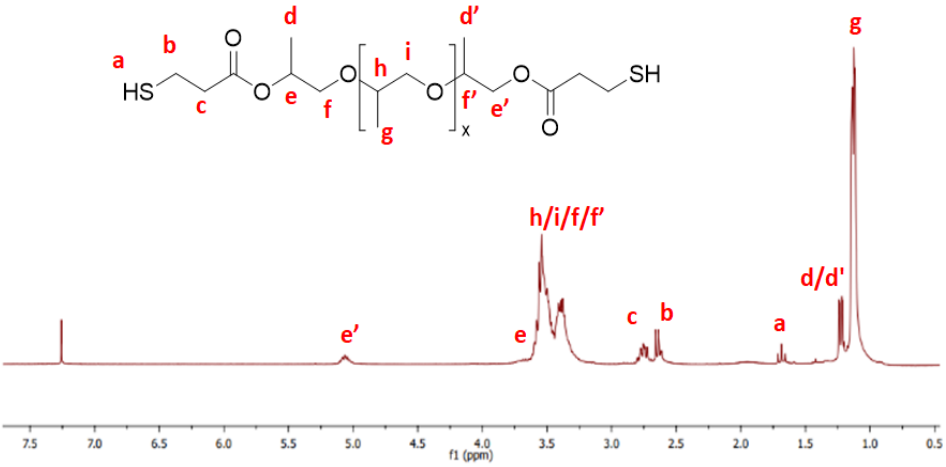

Figure S2. ^{1}H NMR spectra of polymer **P2** in $CDCl_3$.

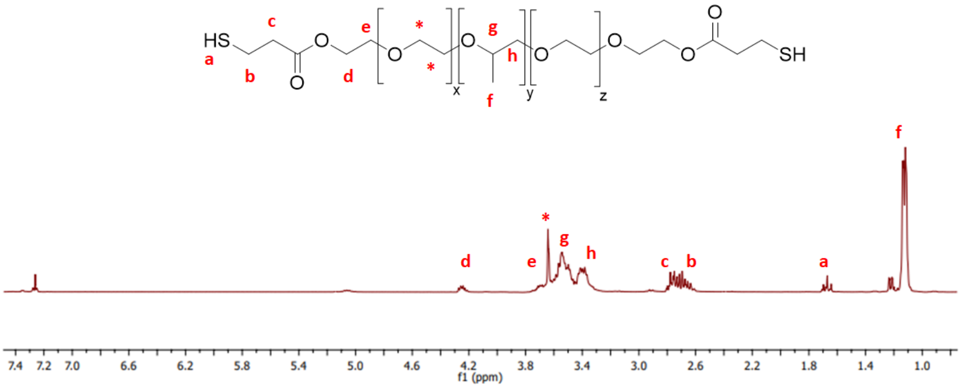

Figure S3. ^{1}H NMR spectra of polymer **P3** in $CDCl_3$.

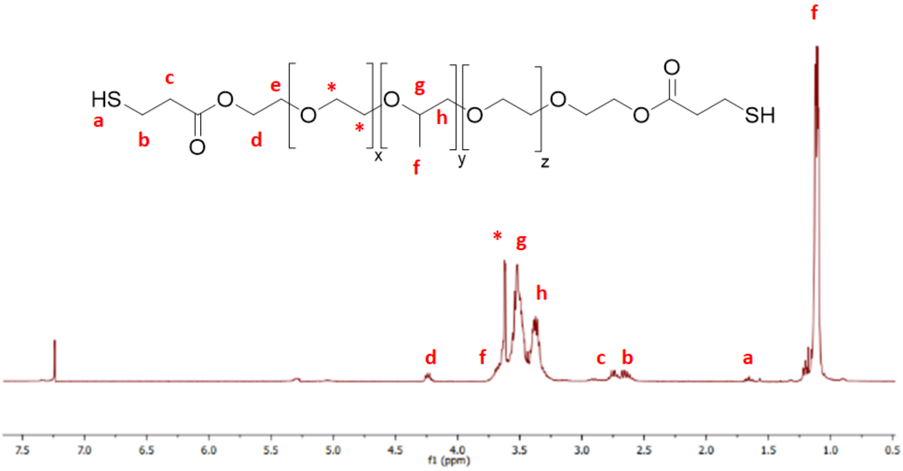

Figure S4. ^{1}H NMR spectra of polymer **P4** in $CDCl_3$.

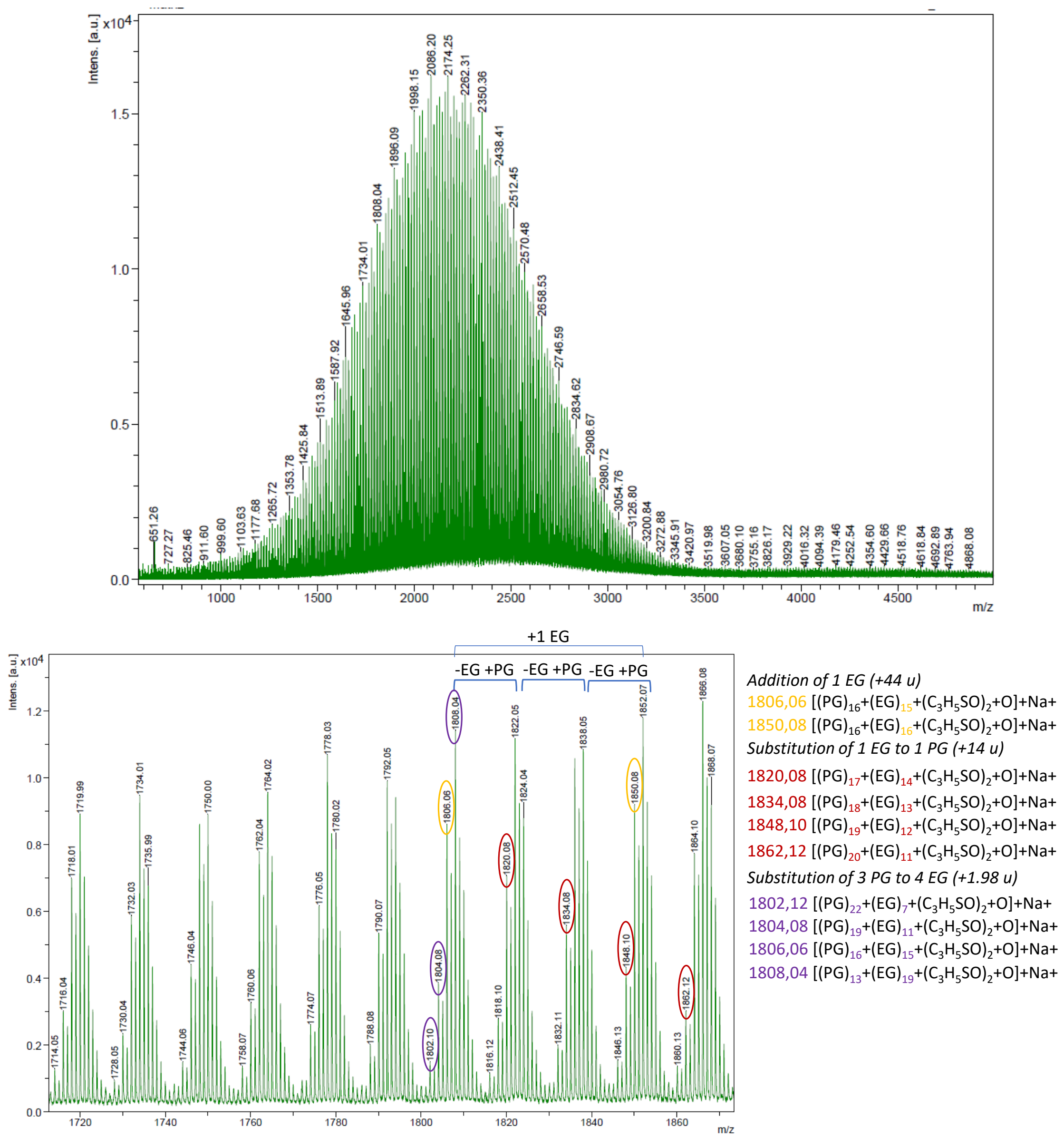

Figure S5. MALDI spectra of polymer **P5** in DCTB and NaI. Top: Full spectrum in the range 500-5000 Da. Bottom: Selected section of the spectrum in the range 1710-1870, with identification of the different peaks, highlighting the complexity of the characterization.
